# Prioritizing interventions to address healthcare worker barriers to reporting adverse events following immunization in Ghana

**DOI:** 10.1016/j.vaccine.2025.127324

**Published:** 2025-05-30

**Authors:** Sharon Laryea, Erin Blau, Alexander Dodoo, Evelyn Addo, Bernice Owusu-Boakye, Kwame Amponsa-Achiano, Naziru Tanko Mohammed, Abena Asamoa-Amoakohene, Jane Gidudu

**Affiliations:** a African Collaborating Centre for Pharmacovigilance and Surveillance, Accra, Ghana; b US Centers for Disease and Control and Prevention, Atlanta, USA; c Expanded Programme on Immunization, Accra, Ghana; d Ghana Food and Drugs Authority, Accra, Ghana

**Keywords:** Reporting barriers, Adverse events following immunization, Frontline immunization healthcare workers

## Abstract

Despite increasing global efforts to enhance vaccine safety, there are still major gaps in the African subregion’s capacity to adequately monitor and respond to vaccine concerns. In 2017 and 2019, two assessments were conducted in Ghana to identify barriers to adverse events following immunization (AEFI) reporting among healthcare workers (HCWs), which revealed multiple barriers and significant gaps in the reporting process, underscoring the need to evaluate approaches to enhance training and support for frontline HCWs. Building upon these assessment findings, in 2021 key immunization safety stakeholders in Ghana met to address the persistent barriers hindering effective reporting practices. Using a feasibility-impact matrix and other qualitative factors, structured discussions allowed stakeholders to identify four innovative strategies to improve AEFI reporting: AEFI reporting toolkit and job aid, HCW protection policy, and new hire training package. Design and implementation of these interventions will allow Ghana to address ongoing barriers to AEFI reporting.

## 1. Introduction

Continuous surveillance of adverse events following immunization (AEFI) ensures the safety of vaccines and provides a means to critically evaluate and communicate vaccine safety findings [1], thereby maintaining public confidence in immunization programs [2]. The World Health Organization (WHO) defines an AEFI as “any untoward medical occurrence that follows immunization and does not necessarily have a causal relationship with the usage of the vaccine” [3]. WHO recommends that countries systematically collect, analyse, and evaluate medically important AEFI in their immunization programs, and this remains increasingly important in low- and middle-income countries (LMICs) due to limited healthcare resources and the higher burden of vaccine-preventable diseases, amidst resource constraints [4,5]. Therefore, robust AEFI surveillance becomes essential in LMICs to identify and mitigate any potential risks associated with immunization programs, ultimately contributing to the prevention of disease outbreaks and the protection of public health. Yet, despite global efforts being made to enhance vaccine safety, there are still major gaps in the African subregion’s capacity to adequately monitor and respond to vaccine concerns. In 2022, less than half (47 %) of countries in the WHO African Region met the new vaccine safety reporting indicator, at least one serious AEFI reported per 1 million total national population per year, a case-based reporting indicator intended to increase accurate AEFI reporting and national surveillance system sensitivity in detecting vaccine safety signals [6].

In 2017 and 2019, two assessments were conducted to identify barriers to AEFI reporting among frontline healthcare workers (HCWs) in Ghana [7–9]. The first assessment, conducted in four regions (Greater Accra, Northern, Upper East, and Volta) in 2017, revealed that only one-third of HCWs who encountered an AEFI reported it, either by filling out an AEFI form or utilizing other existing reporting modes [7]. The study identified multiple barriers contributing to low AEFI reporting among HCWs, including fear of personal consequences, lack of knowledge and training, lack of motivation, and HCWs not believing that an AEFI was serious enough to report. These findings highlighted significant gaps in the reporting process and underscored the need for enhanced training and support for HCWs using innovative approaches. To build on these findings, a follow-up assessment was conducted in 2019 and included a purposive sample of 387 HCWs in the Greater Accra and Ashanti regions [8]. This assessment aimed to identify additional barriers and provide a more comprehensive understanding of the challenges faced by HCWs prior to selecting appropriate interventions. The 2019 study found that while most HCWs had some knowledge of the AEFI surveillance system, this did not necessarily translate into improved AEFI reporting. Additional barriers identified included the unavailability of AEFI reporting tools, over-reliance on caregivers to report AEFIs, caregivers’ failure to report to facilities, fear of victimization for reporting, and lack of feedback when reporting. Furthermore, a significant number of HCWs lacked knowledge in the existing reporting system and when to report an AEFI, indicating that traditional training methods used in Ghana were insufficient in building capacity among frontline HCWs, including reliance on training of trainers and didactic approaches [7].

The assessments revealed persistent challenges that hinder effective AEFI reporting by HCWs, consistent with challenges identified in other countries [10–15], and underscored the need for innovative strategies to address these issues in Ghana. Overcoming these barriers is crucial for effective AEFI surveillance, particularly with the introduction of new vaccines such as those for COVID-19, malaria, and meningitis in Ghana.

This paper presents the outcomes of a stakeholder meeting aimed at identifying innovative strategies to enhance AEFI reporting among frontline immunization HCWs in Ghana. In this meeting participants sought to address the persistent barriers hindering effective reporting practices in the country. This manuscript highlights the discussions, conclusions, and recommendations generated by stakeholders from diverse sectors, emphasizing the critical need for improved AEFI surveillance and reporting systems.

## 2. Methods

### 2.1. Stakeholder meeting

In 2021, a one-day stakeholder meeting was convened by the African Collaborating Centre for Pharmacovigilance (ACC) to review previous assessment findings and propose novel strategies to address barriers in reporting AEFI among frontline HCWs in Ghana. The meeting included 27 key stakeholders representing the U.S. Centers for Disease Control and Prevention (CDC), Ghana Expanded Programme on Immunization (EPI), Ghana Food and Drugs Authority (FDA), ACC Committee on Vaccines and frontline immunization HCWs from the Ghana Health Service (GHS) and non-governmental sector and their supervisors.

### 2.2. Feasibility impact matrix

**Fig. 1.**
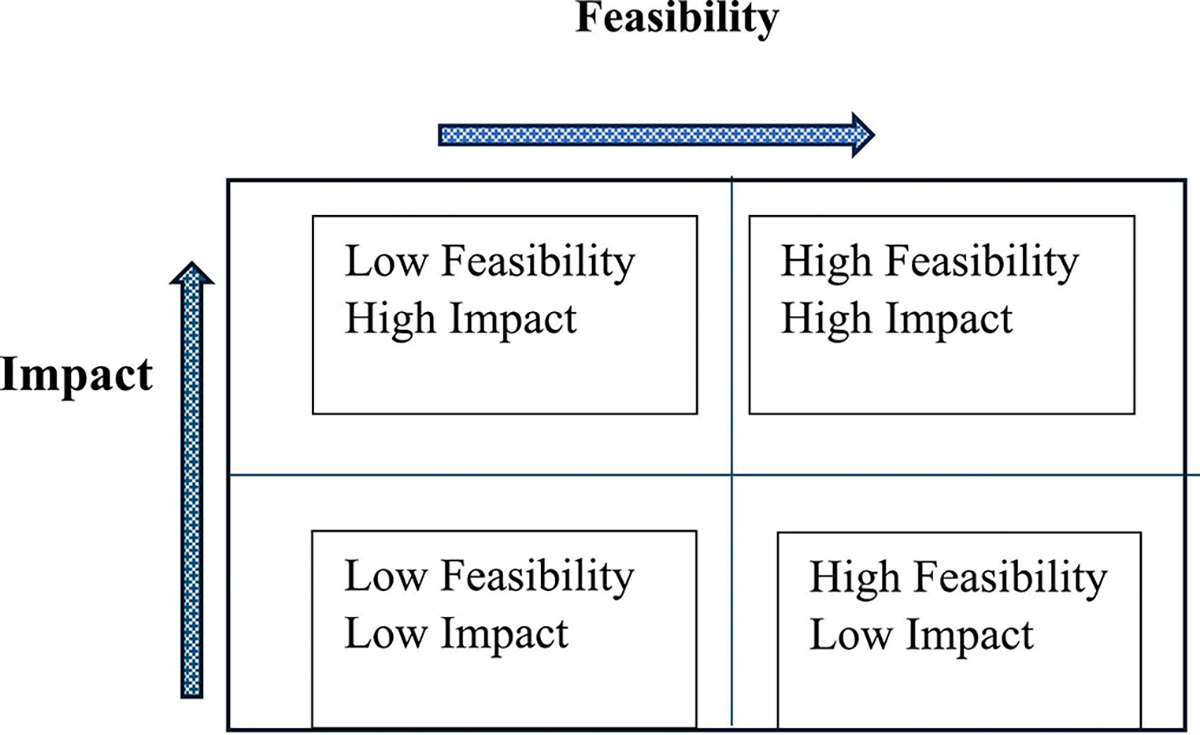
Feasibility Impact Matrix.

A Feasibility-Impact Matrix (FIM) was applied to systematically prioritize potential interventions (Fig. 1). The matrix facilitated a structured deliberation where stakeholders collectively assessed and scored each intervention strategy based on its feasibility and potential impact on improving AEFI reporting.

The FIM categorizes tasks or projects into a 2 × 2 matrix, dividing them into four quadrants based on two primary dimensions [16]:

- Feasibility Scores (1–5, Least-Most Feasible): Stakeholders rated each strategy based on practicality, ease of implementation, resource availability, and stakeholder acceptability.
- Impact Scores (1–5, Lowest to- Highest): Stakeholders assessed the potential positive effects of each strategy on AEFI reporting improvement, considering factors such as enhanced surveillance, increased reporting rates, and improved data quality.

### 2.3. Prioritization of interventions

The total scores obtained from the FIM guided the identification and prioritization of interventions to ensure a focused approach to addressing the identified barriers to AEFI reporting in Ghana. Each potential intervention was scored using the FIM, with total scores derived by combining the feasibility and impact ratings for each intervention. Interventions were then ranked based on these total scores, with higher scores indicating a higher priority for implementation. The process involved a collaborative and consensus-based approach with the multiple stakeholders. Discussions were conducted to gather insights and ensure that the scoring reflected practical experiences and expert opinions. This process allowed stakeholders to collectively identify interventions that were both feasible to implement and had the potential to significantly impact AEFI reporting outcomes. In Ghana, from the initial list of interventions, those with the highest total scores were identified, considering both feasibility and impact. Additionally, the strategic alignment of each intervention, the practicality of implementing them within the designated timeline, and other factors as identified by stakeholder expert opinion were considered.

## 3. Results

The stakeholder consultative meeting facilitated a comprehensive evaluation of proposed interventions aimed at enhancing AEFI reporting by HCWs in Ghana. Using the FIM, interventions were assessed and scored for feasibility and impact (Table 1). In addition to numeric scores, stakeholder deliberations considered other factors such as feasibility of implementation within the available timeline and resources, resource availability, system integration, sustainability, stakeholder acceptance, and the evidence base supporting intervention effectiveness in similar contexts. These factors ultimately guided the final selection of four prioritized interventions to address barriers to AEFI reporting among HCWs: an AEFI reporting toolkit, an AEFI reporting job aid, a HCW protection policy, and a new hire training package (Table 2). These four interventions were chosen based on effectiveness, practicality, and sustainability within Ghana’s healthcare system operational constraints.

## 4. Discussion

AEFI surveillance plays a pivotal role in fostering public trust in vaccination programs. The timely and transparent reporting of AEFIs is crucial, to build public confidence in vaccination efforts especially during new vaccine introductions. HCWs contribute significantly to building and maintaining this trust by promptly and accurately reporting AEFIs. The outcomes of the stakeholder deliberations in our study, highlight the systematic process of selecting interventions to address barriers to AEFI reporting among HCWs in Ghana. The process began with numerous recommended interventions, each assessed using a FIM. Despite the initial quantitative assessment, the final selection of interventions was influenced by two qualitative factors identified during stakeholder deliberations: (1) implementation timeline and resource constraints, and (2) system integration.

Interventions such as “set clear expectations & accountability for supervisors to support HCW, including virtual supervision” and “develop standard operating procedures (SOPs) for reporting,” although highly scored, were deemed more complex and resource intensive. Given the study’s timeline and available resources, these interventions were not prioritized. Instead, the focus was on interventions that could be implemented more readily and with existing resources.

The practicality of integrating interventions into the existing healthcare framework was a significant consideration. Interventions that required substantial changes to current systems, such as “Improve FDA feedback process at every level,” faced potential implementation challenges. The prioritization process favored interventions that could be seamlessly incorporated into existing workflows.

Through the extensive stakeholder inputs, four interventions were prioritized to enhance overall AEFI reporting by HCWs in Ghana. The selected interventions are envisaged to provide significant enhancements over traditional training methods for HCWs in AEFI reporting. Traditional training approaches, often limited to sporadic workshops and basic orientations, lack the consistency and follow-up necessary for effective reporting [10–12]. In contrast, the proposed interventions are expected to offer a comprehensive, standardized approach with continuous support and immediate reference tools, ensuring accurate and consistent practices. These interventions aim to create a supportive environment that mitigates fears of retribution, encourages robust reporting, and ensures new HCWs are thoroughly prepared from the outset. By addressing key barriers such as fear, lack of knowledge, and inadequate training, these interventions are anticipated to improve the overall effectiveness of AEFI reporting and enhance vaccine safety surveillance. Confronted with the obstacle of inefficiently disseminating AEFI training across diverse HCWs and facility levels, the prioritized interventions introduce innovative alternatives to conventional training and orientation approaches. The toolkit and poster in particular, guidance, and capacity-building materials are designed to spread information, change or improve behaviors, or build capacity based on the latest evidence and experience.

Therefore, the next steps involve not only adapting and extending the use of these tools to other countries within the African Region but also evaluating the interventions to assess their applicability and effectiveness in diverse contexts. This includes further evaluation of other countries within the region to ensure that the strategies are adapted appropriately and address the specific needs of each setting.

While stakeholders’ express optimism about the potential benefits of these proposed interventions, there are concerns regarding their implementation and potential impact on healthcare providers’ workloads. A systematic review highlighted that merely developing a toolkit and other such interventions aren’t sufficient to ensure its effective utilization and implementation [17]. Additional efforts and knowledge are necessary to ensure that end users can fully comprehend and take advantage of the potential benefits. Without identifying or addressing critical implementation challenges, these interventions may not be optimally utilized. These perspectives underscore the need for meticulous planning and continuous evaluation to ensure the effectiveness and sustainability of the implementation of the interventions over the long term.

## 5. Limitation

While the meeting included immunization safety specialists and key stakeholders in Ghana, the selection of these four interventions to target AEFI reporting barriers among HCWs reflects the priorities identified by the meeting attendees and may not reflect the priorities of stakeholders in other countries. Likewise, the four interventions chosen were unique to the issues identified in Ghana during earlier assessments and at the time of the meeting. In addition to scoring each intervention, stakeholders also considered the practicality of implementing within a designated timeline, resource availability, system integration, sustainability, and stakeholder acceptance. Others may choose to score certain interventions differently, due to different timelines, resources, systems, and stakeholder input, which would greatly impact what interventions will be prioritized in each country. Lastly, while these findings may be relevant to settings with similar resources and timelines, countries should prioritize conducting assessments to understand unique barriers and facilitators to reporting among HCWs and work to adapt interventions to their specific needs.

## 6. Conclusion

The selected interventions—an AEFI reporting toolkit, an AEFI reporting job aid (poster), a HCW protection policy, and a revised EPI new hire training package—are anticipated to significantly enhance AEFI reporting practices, thereby improving vaccine safety and public health outcomes in Ghana. It is envisioned that these interventions will not only bolster AEFI reporting among frontline immunization HCWs nationwide but will also potentially enhance reporting throughout the African Region. The next steps involve the design and development of these interventions, which will first be implemented in Ghana and subsequently extended to other selected countries in the region.

## Figures and Tables

**Table 1:** Stakeholder-Recommended Interventions to Address Barriers to AEFI Reporting Among Healthcare Workers: Feasibility and Impact Assessment, February 2021.

Recommended Interventions	Feasibility	Impact	Total Score

AEFI reporting Toolkit including an implementation guide, supervisors' action plan template	High (3)	High (5)	8
Create & distribute AEFI Reporting Job aid (Poster)	High (3)	High (5)	8
Set clear expectations & accountability for supervisors to support HCW, including virtual supervision	High (3)	High (5)	8
Develop SOPs for AEFI reporting	Medium-High (2.5)	High (5)	7.5
Implement HCW protection policy	Medium (2)	High (5)	7
Improve FDA feedback process at every level	Medium (2)	High (5)	7
Revise EPI new hire training package	High (3)	Medium (3)	6
Introduce AEFI performance indicators at health facility level	Low (1)	High (5)	6
EPI & FDA simplify AEFI reporting form	Low (1)	High (5)	6
EPI & FDA improve AEFI reporting logistics	Low (1)	High (5)	6
Train supervisors on techniques to give corrective feedback	High (3)	Medium-low (2)	5
Fortify health education & community awareness	Medium (2)	Low (1)	3
Revise preservice curriculum	Medium (2)	Low (1)	3

AEFI: adverse events following immunization

HCW: healthcare workers

SOPs: standard operating procedures

FDA: Food and Drugs Authority

EPI: Expanded Programme on Immunization.

**Table 2:** Stakeholder-Prioritized Interventions and Purposes, February 2021.

Intervention	Purpose

AEFI Reporting Toolkit including an Implementation Guide and Supervisors' Action Plan Template	To provide a comprehensive resource for healthcare workers and supervisors, detailing step-by-step procedures for identifying, documenting, and reporting AEFIs. This toolkit aims to enhance knowledge, streamline reporting processes, and ensure consistent application of best practices across all healthcare facilities.
Create & Distribute AEFI Reporting Job Aid (Poster)	To offer a quick reference guide for healthcare workers, summarizing key points on AEFI identification and reporting. The poster serves as a visual reminder, reinforcing training and encouraging timely and accurate reporting of AEFIs.
Implement HCW Protection Policy	To establish clear guidelines and protections for healthcare workers who report AEFIs, thereby addressing fears of personal consequences and victimization. This policy aims to foster a supportive environment where healthcare workers feel safe and motivated to report AEFIs.
Revise EPI New Hire Training Package	To update the training curriculum for new hires in the EPI to include comprehensive education on AEFI reporting. This revision ensures that all new healthcare workers are well-equipped with the necessary knowledge and skills to effectively identify and report AEFIs from the start of their careers.

## Data Availability

No data was used for the research described in the article.

## Abbreviations

ACC: African Collaborating Centre for Pharmacovigilance
AEFI: adverse events following immunization
CDC: U.S. Centers for Disease Control and Prevention
EPI: Expanded Programme on Immunization
FDA: Food and Drugs Authority
GHS: Ghana Health Service
HCWs: healthcare workers
LMICs: low- and middle-income countries
SOPs: standard operating procedures
WHO: World Health Organization
